# Postgraduate Study

**Published:** 1919

**Authors:** J. M. Fortescue-Brickdale

**Affiliations:** Physician to the Bristol Royal Infirmary


### POSTGRADUATE STUDY.

BY

J. M. Fortescue-Brickdale, M.A., M.D., M.R.C.P.,

*Physician to the Bristol Royal Infirmary.*

Years ago at Guy's Hospital it used to be the custom of the Pupils' Physical Society to ask an old Guy's man once a year to come and give an address at the opening meeting of the session. The lecturers were not, as far as I remember, usually specialists or consultants, but eminent general practitioners, and the practice was certainly a good one, and the lectures often of considerable valne, especially to senior students. On one occasion the subject chosen by the lecturer, who was a well-known and successful medical man, was, " How to remain a student through life." It may be read by anyone who cares to look up the back numbers of the *Guy's Hospital Gazette*, somewhere in the nineties. I remember at the time, as most of us were engaged in fighting the examination fiend, the subject appeared to have a touch of irony, and some " chronics " openly asserted that they knew more about it than the lecturer.

But I do not think now that any medical man, general practitioner, consultant or even the most special of specialists, would consider the condition of perpetual studentship at all easy to attain. We all tend to get into grooves and to be contented with the methods we have always used and the knowledge we seem always to have possessed ; and even the exercise of teaching the unfledged student does not always entirely ensure that openness of mind and habit of scientific inquisitiveness which is so essential to real progress.

In one sense all medical men must remain students through life, for all must study their cases ; but for the

General Practitioner one difficulty seems to be that he has to spend a great deal of time in studying his patients, and cannot therefore give sufficient time to the study of disease in the scientific sense. But advance in medicine, to be effective, must be made on a wide front. Isolated attacks, however brilliant and well planned, do but little to compel a general retirement on the part of the enemy. In the present state of things the opportunities for countless valuable observations are lost, because the men qualified to make them have neither the time nor the necessary apparatus and equipment. There are of course exceptions. Jenner, as we all know, was a country practitioner, and there have recently been other examples of valuable work done in the course of a very busy practice. But we have to legislate for the average man and not for the exceptional man, and that is why it seems to me that some steps should be taken to make it possible for the many men who start well qualified in their profession to continue those studies which they have begun in the wards of their hospital or the laboratories of their medical school.

Minute studies of rare or exceptional diseases may bring fame, but are they always of the same value to science and humanity as careful investigations on the common and everyday conditions which are dealt with almost exclusively by the General Practitioner? If, for instance, we knew as much about measles as we do about myxœdema, and could treat it with the same success and certainty, what an enormous difference it would make to our mortality statistics at the end of only one year.

Then, again, how much do we know of the slight and insidious beginnings of disease, of familial and hereditary tendencies, of the consequences of slight ailments? These things can only be observed by the General Practitioner, and are a work extending over many years. What opportunity has he for duly recording and analysing his observations, even if he has time for detailed observations of any kind?

I need not perhaps labour these points, but what I wish to emphasise is that immense possibilities lie open to the General Practitioner if means can only be devised for enabling him to use them fully.

I will now pass on to outline what in my opinion are some of the ways in which organised postgraduate study may help towards enabling the General Practitioner to make use of the material and experiences which are round him for the advancement of medical science and the benefit of humanity. It seems to me that there are five conditions which, if they could be fulfilled, would go some way towards realising this ideal. Means should be forthcoming to enable the General Practitioner to—

*Firstly.*—Keep abreast of the general scientific advances of the time.

*Secondly.*—Keep in touch with the outstanding clinical and pathological observers of the city, district, or state.

*Thirdly.*—Command expert assistance in the way of laboratory and pathological investigations.

*Fourthly.*—Keep and study his systematic records.

*Fifthly.*—Most important and most difficult condition of all, obtain sufficient time to do all these things.

These five points may now be considered in detail, as the success of their practical realisation depends on the scope which is assigned to them, and methods by which they are put into practice.

1. To keep abreast of general medical and scientific knowledge is not easy. It is not done by merely skimming through a number of medical journals, whether English or foreign, or by the study of epitomes and abstracts. Much that is printed is far from being knowledge, and the time required for the process of sifting the valuable material from the dross is too great for most men in general practice. It is here that courses of postgraduate instruction may be of considerable value. Hospital physicians and surgeons and pathologists must always to some extent be specialists and in a position to appraise the value of scientific communications on the subjects or branches of knowledge in which each one is specially interested. Without giving undue weight to specialism, all in such a position should be able to embody some results of critical study in lectures and demonstrations.

2. By the second condition I mean to imply that in all centres of medical education there are (or should be) men who stand out conspicuously as clinical observers and teachers. To enable these men to use their powers to the best advantage, they should be given such a position both academically and financially that they can devote the greater part of their time to organising and inspiring the teaching and researches of their colleagues, as much without as within the walls of their university and the wards of their hospital. The great gifts of accurate observation and critical estimation, the power of co-ordinating results and detecting fallacies of method, are not given to all alike, and those men who are most endowed with these faculties should be able to diffuse them over as wide a circle as possible ; to do this they must be relieved of much of the routine of practice, and brought into touch with all those who are engaged in medical studies, whether undergraduate or postgraduate.

Moreover, I would suggest that in any good scheme of postgraduate study provision should be made for professors and clinicians from other centres of learning to give occasional lectures, demonstrations or courses of instruction.

3. By expert laboratory assistance I mean something more than staining tubercle bacilli in sputum, or estimating urea in the urine. If the General Practitioner is to treat his patients scientifically and to be in a position to advance the knowledge of diagnosis and treatment, he must be kept in personal touch with the pathologists, and by means of courses of instruction be informed of the nature of the investigations which are likely to lead to good results, and the precautions to be taken in obtaining material for them. A general knowledge of recent laboratory methods, of their applicability to given cases, and of results obtained elsewhere are essential if the clinician and the pathologist are to work together in fruitful concert.

4. So far it would seem that the three conditions hitherto outlined can be met by courses of organised instruction at a well-equipped centre. Towards realising the fourth condition, and also as a supplementary measure as regards the other three, I make the following suggestions.

The systematic keeping of records is largely a matter of training and habit, and this can, of course, best be acquired in the wards of a hospital. As applied to the General Practitioner, this may be brought about in two ways, not necessarily antagonistic, but rather complementary to each other.

The first way is by the establishment of clinical assistantships for men in general practice, to be held for a limited time and involving definite hours of attendance. Preferably, I think, this should be done in postgraduate wards and out-patient clinics, where the method of work could be specially adapted to the needs of the practitioner.

The other is by organising General Practitioners' Hospitals. These should be furnished with the staff and equipment of an ordinary general hospital, and should be provided with resident medical officers. Probably special departments would not be required, and the work would be mainly medical rather than surgical. Here the practitioner could admit his own cases, not necessarily without payment, and could observe and record his observations. The cases would, as a rule, include the more common and less severe types of disease and injury which badly need extended and careful observation. By linking these hospitals with the central clinical institution the practitioner could secure the co-operation and advice of any member of the consultant teaching staff if he wished to do so, and by affiliation to the University facilities for pathological investigations could be provided.

5. This, as I have already stated, is by far the most important and difficult problem in the whole question of postgraduate study. The following suggestions must be considered as a partial solution, for everything depends on a careful working out of detail, and this is hardly possible in a short paper like the present. Two considerations present themselves : firstly, to the General Practitioner as much as to any other business man time is money ; if he is to take time from his business and devote it to study, it is difficult to see how he can fail to be a loser financially. Of course increased efficiency may in some cases bring increased income, but not in all by any means. Secondly, how can the necessary attendance on the sick be carried on if an appreciable amount of the time of many of the doctors is taken up by other avocations ?

As regards the first of these considerations, I think that the State should among its various benefactions endow post-graduate education, which must in the end benefit the whole mass of the population. A certain number of exhibitions or bursaries should be founded for medical men who desire to undertake postgraduate study ; they might be awarded by the local University or by a specially constituted committee representing the University and Municipal authorities, with perhaps some elected representatives of the practitioners themselves. The actual value of the bursary need not be large, but should be enough to compensate the postgraduate student for loss of business while engaged on courses of study, and should only be tenable during the continuance of the course.

With respect to the second consideration, it must be remembered that a certain proportion of every doctor's time is taken up by visits that need not be paid, and further that want of consideration for his time on the part of the public is not infrequent. During the war the public has probably to some extent been forced to appreciate this fact, so that now is an excellent time for seeing that the lesson is not forgotten. But apart from this, it seems possible that some system of co-operation could be devised among the medical men of a district or area whereby one or more of them could devolve his work on the others during the hours he devoted to postgraduate studies. This system when adopted on a sufficiently wide scale need not involve much financial loss ; fees earned in the doctor's absence should be credited to him entirely, and the system being mutual would be fair to all. The public soon grows accustomed to any generalised system, and in the end, of course, would benefit by the increased efficiency of the medical service.

To give effect to any system or scheme such as has been outlined in this paper a propaganda is necessary. Possibly this could be furthered by the establishment of a " Union for Postgraduate Studies," with a nominal subscription, which could discuss details from the General Practitioner's point of view, gain publicity for the idea, and enlist the co-operation of the mass of the profession, without which very little advance can be made.

